# Water-mediated crystallohydrate–polymer composite as a phase-change electrolyte

**DOI:** 10.1038/s41467-020-15415-5

**Published:** 2020-04-15

**Authors:** Ziyang Tai, Junjie Wei, Jie Zhou, Yue Liao, Chu Wu, Yinghui Shang, Baofeng Wang, Qigang Wang

**Affiliations:** 1 Shanghai Key Laboratory of Materials Protection and Advanced Materials in Electric Power, Shanghai University of Electric Power, Shanghai, 200090 China; 2 School of Chemical Science and Engineering, Tongji University, Siping Road 1239, Shanghai, 200092 China

**Keywords:** Gels and hydrogels, Composites

## Abstract

With the world’s focus on wearable electronics, the scientific community has anticipated the plasticine-like processability of electrolytes and electrodes. A bioinspired composite of polymer and phase-changing salt with the similar bonding structure to that of natural bones is a suitable electrolyte candidate. Here, we report a water-mediated composite electrolyte by simple thermal mixing of crystallohydrate and polymer. The processable phase-change composites have significantly high mechanical strength and high ionic mobility. The wide operating voltage range and high faradic capacity of the composite both contribute to the maximum energy density. The convenient assembly and high thermal-shock resistance of our device are due to the mechanical interlocking and endothermic phase-change effect. As of now, no other non-liquid electrolytes, including those made from ceramics, polymers, or hydrogels, possess all of these features. Our work provides a universal strategy to fabricate various thermally manageable devices via phase-change electrolytes.

## Abstract (web summary)

Here the authors report composite electrolytes combining polymer chains and hydrated salts with a similar bonding structure to that of natural bones. The design breaks the trade-off between strength and ionic mobility of solid electrolytes and allows for good electrochemical performance in supercapacitors.

## Introduction

Organic–inorganic composite materials have been widely used in many applications, including biomaterials^1–4^, catalysts^5–7^, automotive materials^8,9^, electronic device^10–14^, and building materials^15,16^. The major challenge for composite material properties is their interfacial compatibility between organic and inorganic components^17^. A graft polymerization and covalent modification of inorganic components can enhance their surface solubility with a polymer matrix, which can partly overcome the interfacial compatibility problem^18–22^. Relative to complicated designs based on the principle of similar compatibility, an evaluation of nature shows exquisite designs of tough bone and seashell connected by crystal-surface-bound water as a transition phase to connect the organic and inorganic components that have a large surface energy difference^23–25^. In ideal bone tissue, 65 wt% inorganic hydroxyapatite provides strength and hardness and 25 wt% organic collagen provides ductility and toughness^26,27^. The key is the 10 wt% water, which acts as an interfacial agent to coordinate the hard inorganic layer and the flexible polysaccharide protein layer^25,28,29^. In the course of a phase-changing composite study, we notice that the thermal mixing of hydrated salts and polymer chains forms viscous gels and transfers them into temperature-dependent composites. This observation has motivated us to search for why crystallohydrates can combine with hydrophilic polymers to form tough composites during a heating-cooling procedure. The water in crystallohydrate should be the key component for the interfacial transition from an inorganic crystallohydrate to hydrophilic polymer chains. As indicated by natural bones, water molecules can be further involved in a proton chemical exchange with mineral surface species^25,30^. These phenomena encourage us to study the ionic mobility and other properties that originate from phase-changing inorganic components.

Due to the risk of easily leaking liquid electrolytes, it is urgent to solve the safety demand of wearable electronics^31–33^, and numerous non-liquid electrolytes, including ceramic, polymer, and hydrogel electrolytes, have been developed^34–38^. However, the narrow operating voltage range and weak mechanical strength of traditional hydrogel electrolytes have limited their practical applications; in addition, ceramic and polymer electrolytes have been hindered by poor interfacial compatibility and low ionic conductivity, respectively^39,40^.

In this work, a crystallohydrate phase is introduced into a polymer matrix to remedy these shortcomings. The three-phase skeletal structure consisting of an intrinsic high-strength crystallohydrate phase, a tough polymer phase, and an intermediate water layer affords these phase-change composite electrolytes (PCCEs) excellent mechanical properties and high ionic conductivity. In addition, a recrystallization of an inorganic salt can suppress the electrochemical activity of water and broaden the operating voltage of electrolytes attributable to the strong recombination of hydrated crystals with water, such as the solvation sheath in “water-in-salt” electrolytes^41,42^. Furthermore, the composite with hydrated salt can reversibly store and release large amounts of latent heat during the solid–liquid phase transition^43–45^, which also endows the composite with processability similar to plasticine in dailylife.

## Results

### Material synthesis and characterization

**Fig. 1:**
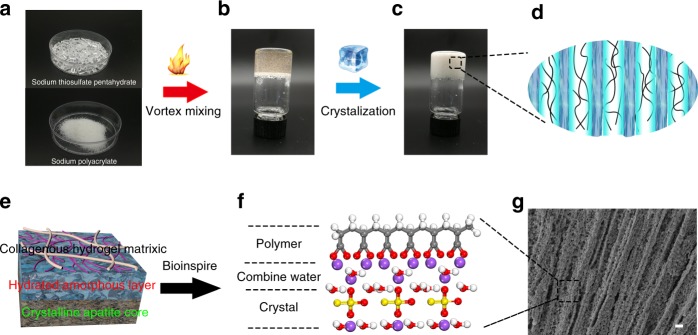
Schematic illustration of the material synthesis. **a** Optical image of sodium thiosulfate pentahydrate and PAANa power. **b** Optical image of the composite in the castable state. **c** Optical image of the composite in the crystalline state. **d** Schematic diagram of the structure of the composite. **e** Schematic diagram of the interface in bones. **f** Molecular model of composite. **g** Scanning electron microscope of the ST crystals within the polymer network. Scale bar, 1 μm.

In this work, a crystallohydrate–polymer composite with high-strength, thermal-shock resistance and high ionic conductivity was prepared by an extremely simple thermal-mixing method (Fig. 1). The precursor contained only two components: sodium thiosulfate (ST) pentahydrate and sodium polyacrylate (PAANa) (Fig. 1a). First, the ST was dissolved at 55 °C and uniformly mixed with PAANa powder at different mass ratios (2:1, 4:1, 6:1, 8:1, and 10:1) for 1 min. The mixtures were then heated at the same temperature for 1 h, yielding crystallohydrate–polymer composite samples in a castable state (Fig. 1b). Then, under the perturbation of surface pressure by a glass rod, the composite containing the self-dissolving ST solution began to crystallize. Finally, the phase transition was complete, and a crystallohydrate–polymer composite was formed with parallel stalactite-like ST crystals (Fig. 1c). The crystallohydrate–polymer composite was recorded as “PAANa-ST_*x*_,” where “*x*” represents the mass ratio of ST to PAANa. For example, “PAANa-ST_6_” means the sample was prepared using a mass ratio of ST to PAANa of 6.

The crystallization process of the self-dissolving ST solution in the composite is shown in Supplementary Movie 1. In all samples, the broad ST crystals began to grow from the contact surface to the bottom after perturbing the composite surface, and stalactite crystals were arranged in parallel (Fig. 1d). The diameter of the stalactite crystals was ~1 μm, which was observable by scanning electron microscope characterization (Fig. 1g), proving that the crystallohydrate–polymer composite was successfully prepared. XRD and FT-IR analysis showed that the crystallohydrates within the polymer network were ST crystals. (Supplementary Figs. 1 and 2)

### Synergistic mechanical properties and plasticity due to crystal-surface-bound water

**Fig. 2:**
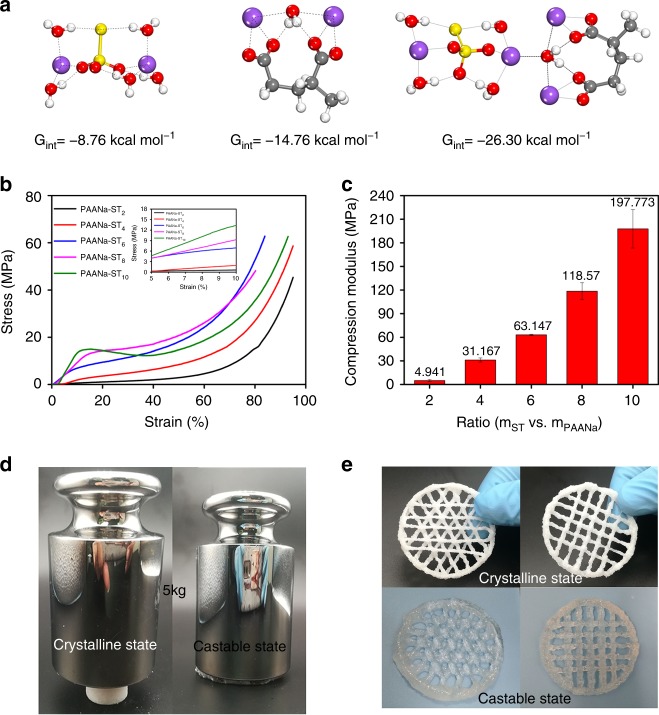
Mechanical properties of composite materials. **a** Relative free energies at 328 K of the water in ST versus that in PAANa and interaction energy between ST and PAANa at 298 K. **b** Compressive curves of the composites with different mass ratios of ST in crystalline state. **c** Compression modulus of the composites with different mass ratios of ST in the crystalline state. **d** Hardness comparison of the composites in the castable and crystalline states. **e** Optical image of 3D printed composite.

The crystalline water from the ST crystal transformed into free water during the heating process. According to a molecular simulation using a Gaussian method (Fig. 2a and Supplementary Table 1), the interaction energy (Eint) between a PAANa molecule and H_2_O was −14.76 kcal mol^−1^, which was greater than Eint (−8.76 kcal mol^−1^) between Na_2_S_2_O_3_·4H_2_O crystals and H_2_O. Therefore, PAANa molecules obtained a water molecule from the ST crystal, and due to water mediation, the Eint between the PAANa polymer and ST crystals was −26.30 kcal mol^−1^. This resulted in a tight interaction between ST and PAANa and formed a three-phase structure that was similar to bones during crystallization (Fig. 1e, f). Based on hydrogen bonding, the crystal-surface-bound water layer in the system provided a synergistic relationship between the hardness of the ST crystal phase and the toughness of the polymer phase, especially when the mass ratio of ST and PAANa was in a range of 2–6. These crystallohydrate–polymer composites exhibited significantly high strength under pressure because of the compact crystal columns arranged in parallel (Fig. 2d). With an increasing mass ratio (ST/PAANa) from 2 to 10, the compression modulus of the composite in the crystalline state was dramatically improved beyond our expectation (Fig. 2b, c). Samples with mass ratios of 6 and above were not broken by the maximum pressure measurable by our equipment. At an ST to PAANa mass ratio of 8, the crystallohydrate–polymer composite demonstrated high strength, but lacked toughness, and the low polymer content could not coordinate with the rigidity of the crystal. The compression modulus of PAANa-ST_6_ was increased by 52,000 times (from 0.0012 MPa in the crystalline state to 63.15 MPa in the castable state) due to the enhancement of the ST crystal compared with that of the uncrystallized sample (Fig. 2c and Supplementary Fig. 3). Although the PAANa-ST_10_ sample lacked toughness, its compressive modulus reached 197.7 MPa, which was ~820,000 times that of the uncrystallized PAANa-ST_10_ sample (0.24 kPa). Similar to PAANa-ST_6_, the PAANa-DPDH_6_ and PAANa-NaAc_6_ samples, prepared in the same manner but with the use of disodium phosphate dodecahydrate (DPDH) and sodium acetate (NaAc), respectively, have compressive moduli of 0.12 and 63.8 MPa (Supplementary Fig. 4), respectively. Therefore, these crystallohydrate–polymer composite materials have excellent mechanical properties. In addition, by adjusting the temperature to control the dissolution and crystallization of ST, these crystallohydrate–polymer composites could be freely converted between rigid (crystalline) and flexible (castable) states (Supplementary Fig. 5). The above property will facilitate the 3D printing of electrolytes (Fig. 2e) and will bring great convenience to electrolyte reprocessing and large-scale industrial production.

### Water-mediated ion movement

**Fig. 3:**
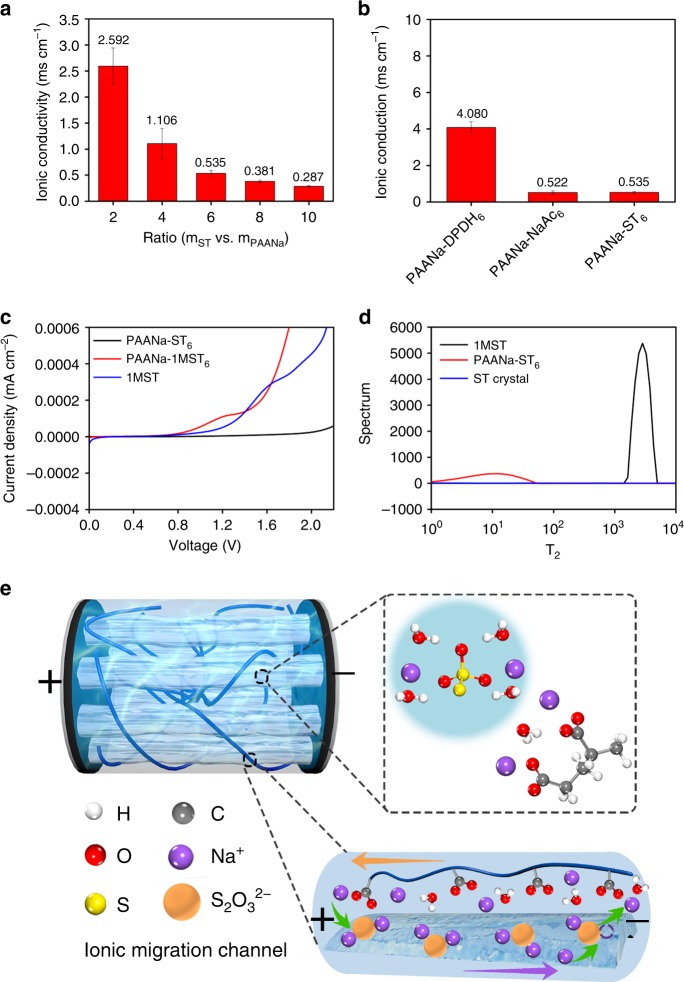
Water-mediated ionic movement. **a** Ionic conductivities of the composites with different mass ratios of ST at room temperature. **b** Ionic conductivities of PAANa-DPDH_6_, PAANa-NaAc_6_ and PAANa-ST_6_ sample. **c** Linear sweep voltammetry curves of the traditional liquid electrolyte with 1 M Na_2_S_2_O_3_, the PAANa gel electrolyte with 1 M Na_2_S_2_O_3_, and the PAANa-ST_6_ composite electrolyte. **d** Low-field nuclear magnetic resonance inversion curves of water signals in 1 M ST, ST crystal, and PAANa-ST_6_ samples. **e** Schematic illustration of the ion migration channel built by zwitterionic groups under an external electric field.

Similar to the contribution of bound water to ionic conduction in bones, the crystallohydrate–polymer composite could be used as an electrolyte in a supercapacitor without adding any external electrolyte salt. The above result is possible due to the presence of water layers that contain a large amount of active ions and the high ionic conductivity of the crystallohydrate–polymer composite. Based on a first-principles calculation (Supplementary Table 2) of the interaction between ST and PAANa, the binding energy of ST to sodium ions in the PAANa system was reduced from 6.34 and 6.56 eV to 6.30 and 6.34 eV, respectively. The ionic conductivity of the crystallohydrate–polymer composite with different mass ratios of Na_2_S_2_O_3_·5H_2_O was measured by electrochemical impedance spectroscopy (EIS) at room temperature (Fig. 3a). The ionic conductivity of the PCCE decreased as the mass ratio of Na_2_S_2_O_3_·5H_2_O increased. The lowest ionic conductivity value was 0.28 mS cm^−1^, which was still much higher than the ionic conductivity of pure ST crystals (~3.42 × 10^−6^ S cm^−1^). The ionic conductivities of the PAANa-DPDH_6_ and PAANa-NaAc_6_ samples were 4.08 and 0.53 mS cm^−1^ (Fig. 3b), respectively, which were both higher than those of most solid polymer electrolytes (10^−6^–10^−3^ S cm^−1^) and ceramic solid electrolytes (10^−7^–10^−3^ S cm^−1^)^34,35^. The latter also had very high mechanical properties. The molecular structure of Na_2_S_2_O_3_·5H_2_O included five crystal waters, which meant that a large amount of free water would become crystal water with a high binding energy during crystallization, thus resulting in a decrease in ion mobility and ionic conductivity. The above observation explained the negative correlation between ionic conductivity and the mass ratio of ST. The electrochemical stability window of three ST-based electrolytes as electrolyte salts (including 1 M ST solution, 1 M ST gel electrolyte, and a PAANa-ST_6_ sample) was evaluated by linear sweep voltammetry (Fig. 3c). The conventional aqueous liquid electrolytes and aqueous gel electrolytes had a voltage window of only ~1 V. In contrast, there was a higher voltage window in the PCCE based on the crystallohydrate–polymer composites mainly because these PCCEs had a unique three-phase structure. The high-temperature free water could be re-fixed to the bound water layer, which contained a large binding energy, by recrystallization at room temperature. As shown in Fig. 3d, the binding energy of water in traditional aqueous electrolytes, namely, PCCE and pure ST crystals, was compared by low-field nuclear magnetic resonance, which is based on the difference in relaxation time between free and bound water (including crystal water). The water binding energy of the PCCE prepared by the ST self-dissolving recrystallization method was much higher than that of the ordinary aqueous solution, and lower than that of the crystal water in the pure ST crystal. In addition, the theoretical calculation indicated that the binding energy of water in a saturated aqueous solution of ST was only 0.27 eV, which was much lower than the binding energy of crystal water (0.51 eV) and crystal-surface-bound water (0.45 eV) in the PCCE, thus confirming the above conclusion. Bound water in the PCCE based on the hydrated salt had a high binding energy and was more difficult to ionize. Therefore, it had a high electrochemical stability window. Increased sodium ion activity is one of the important reasons for the improved ion conductivity of the PCCE. According to the theoretical calculations above, one molecule of water would be attracted to the ST system (Fig. 3e), such that the surface of the crystal column that was in contact with the PAANa polymer chain formed a sedimentary dissolution equilibrium. In addition, the sodium ion “A” in the PAANa system was freed in the ion channel after the activity was increased, thus disturbing the equilibrium of the precipitate on the surface of the Na_2_S_2_O_3_·5H_2_O crystal column. Consequently, an excess of sodium ions “B” was generated in the crystal column dissolution equilibrium and replaced the sodium ion “C” in the ST crystals on the adjacent surface. Then, the generated free sodium ions “C” formed a “jumping conduction” phenomenon under the action of an electric field force, in which the sodium ions that occurred in the “jumping conduction” phenomenon always moved from the cathode to the anode (Fig. 3e). As expected, in the case of a larger proportion of polymer, more hopping occurred, which led to an increase in ionic conductivity. The above phenomenon further explained the negative correlation between the salt concentration and ionic conductivity that had also been reported in the work of Wei et al.^46^. The high activity caused by the “jumping conduction” phenomenon and the presence of bound water in the PCCE system improved the low ionic conductivity of traditional solid polymer electrolytes, and the use of the hydrated crystalline salt provided similar strength to traditional inorganic electrolytes. Thus, the comprehensive performance of the composite electrolyte developed in this work was an objective improvement over conventional solid electrolytes (Fig. 3d).

### Electrochemical performance of the solid-state supercapacitor

**Fig. 4:**
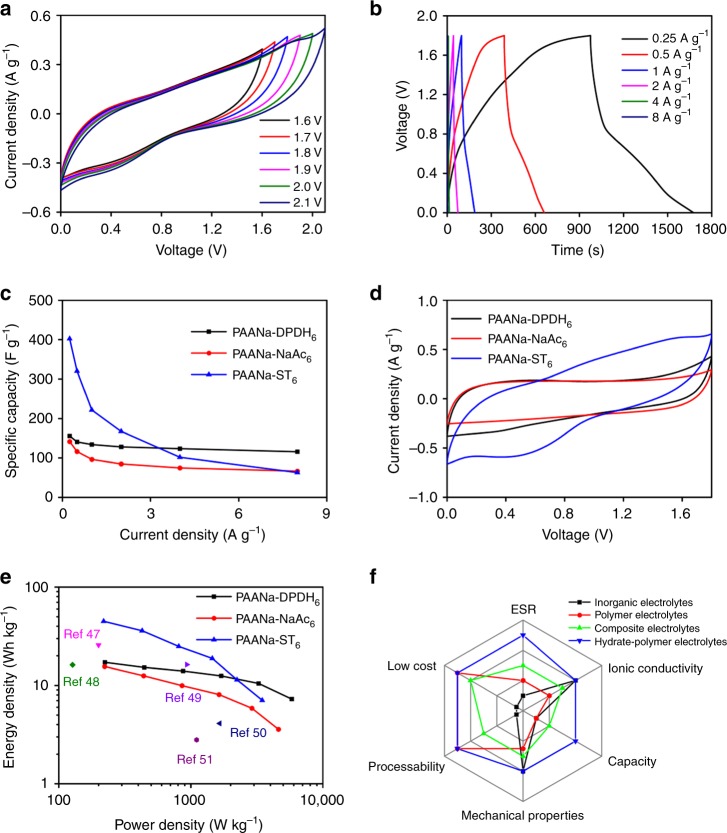
Electrochemical performance of PCCEs-based supercapacitors. **a** Cyclic voltammetry curves of the PAANa-ST_6_-based supercapacitor with different cut-off voltages from 1.6 to 2.1 V. **b** Galvanostatic charge–discharge curves of the PAANa-ST_6_-based supercapacitor with different current density. **c** Specific capacitance of the supercapacitors with different hydrate–polymer composite electrolytes at various current densities. **d** Cyclic voltammetry curves of supercapacitors based on the PAANa-ST_6_, PAANa-DPDH_6_, or PAANa-NaAc_6_ composites at a sweep speed of 5 mV s^−1^. **e** Ragone plot of the supercapacitors with different electrolytes. **f** The radar plots of the comprehensive performances of different electrolytes.

**Fig. 5:**
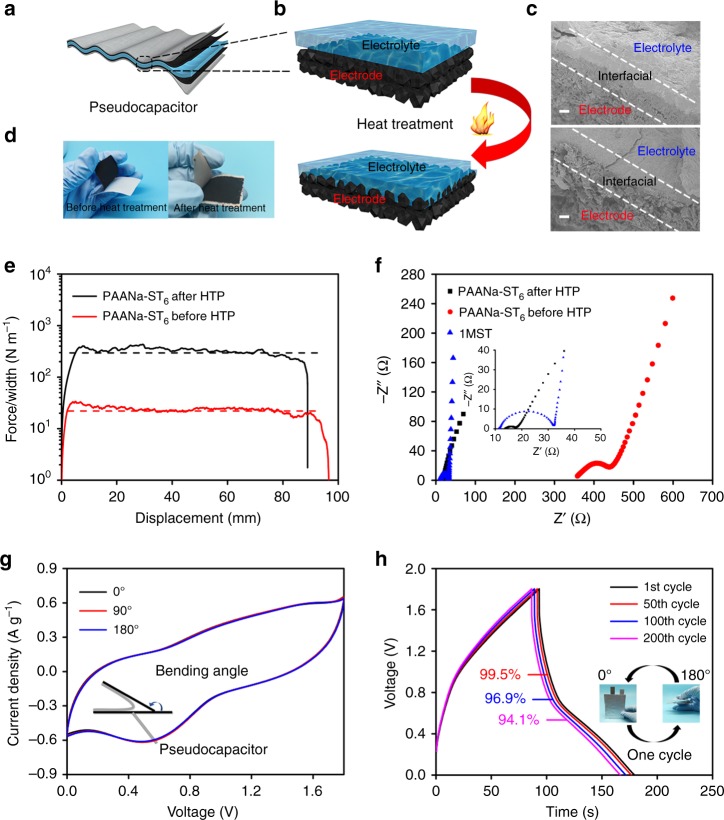
Measurement of electrode–electrolyte interface. **a** Schematic diagram of a flexible capacitor containing active material, current collector, and electrolyte. **b** Electrode–electrolyte interface bonding diagram before (upper panel) and after (lower panel) heat treatment. **c** Scanning electron microscope of the interface between electrode and electrolyte in the capacitor before (upper panel) and after (lower panel) heat treatment. Scale bar, 20 μm. **d** Optical image of the interface between electrode and electrolyte in the flexible capacitor before and after heat treatment. **e** Curves of peeling force per width of the PAANa-ST_6_ sheet and carbon cloth sheet. **f** Nyquist plots of the supercapacitors with different state electrolytes. **g** Cyclic voltammetry curves of the PAANa-ST_6_-based supercapacitor at different bending angles. **h** Galvanostatic charge–discharge curves of the PAANa-ST_6_-based supercapacitor after different bending cycles.

The PAANa-ST_6_ sample was selected as the electrolyte for a supercapacitor due to its mechanical strength and ionic conductivity. Commercial activated carbon powder (YP 80F) was selected as the electrode material. Cyclic voltammetry (CV) and galvanostatic charge–discharge (GCD) with cut-off voltages ranging from 1.6 to 2.1 V were used to determine the proper operating voltage of the PCCE-based supercapacitor. The CV curve was approximately symmetrical when the cut-off voltage did not exceed 1.8 V (Fig. 4a); in contrast when the potential was higher than 1.8 V, the forward current was significantly increased. Correspondingly, the charge and discharge times in a range of 1.6–2.1 V of the GCD (Supplementary Fig. 6) were approximately equal. The above results demonstrate that the ideal operating voltage for supercapacitors with PCCE was 1.8 V. Since a redox-type ST was used as the hydrated salt of the system, the electrolyte had a faradic capacitance that provided a high energy density. Conventional symmetric supercapacitors based on activated carbon typically have a specific capacitance of only 50–200 F g^−1^. The supercapacitor based on PAANa-ST_6_ had a high specific capacitance of 406 F g^−1^ at a current density of 0.25 A g^−1^ and still had 62 F g^−1^ at 8 A g^−1^ (Fig. 4b, c). The supercapacitors based on PAANa-DPDH_6_ and PAANa-NaAc_6_ without faradic capacitance only had specific capacitance of 134 and 96 F g^−1^ at a current density of 1 A g^−1^, respectively (Fig. 4c and Supplementary Figs. 7 and 8). The above results were less than the specific capacitance of the PAANa-ST_6_-based supercapacitor at the same current density (221 F g^−1^). Similar results could be seen from the CV curve (Fig. 4d). The difference was derived from the faradic capacitance provided by the S_2_O_3_^2−^/S_4_O_6_^2−^ redox pair. In addition, among the three supercapacitors, the PAANa-ST_6_-based supercapacitor exhibited an excellent energy density of 42.7 Wh kg^−1^ at a power density of 220 W kg^−1^ (Fig. 4e), which was much larger than that of the supercapacitors based on PAANa-DPDH_6_ (17.2 Wh kg^−1^) or PAANa-NaAc_6_ (15.6 Wh kg^−1^). Furthermore, the PAANa-ST_6_-based supercapacitor offered better performance than most aqueous supercapacitors in terms of energy density because of its higher operating voltage and the hydrated salts containing faradic capacitance in the PCCE^47–51^. The comprehensive performance of the composite electrolyte developed in this work was an objective improvement over conventional solid electrolytes (Fig. 4f)^31,39,40^. Since the PCCE was very hard, the capacitor (Fig. 5a) was assembled in a flexible state before its crystallization, and heated in a 55 °C oven for 12 h before sealing with an MRX-CP60 button battery processor to avoid the problem of excessive interfacial resistance between the electrodes and electrolyte interfaces; the above often occurs in traditional solid electrolytes. The PCCE developed in this study had strong adhesion to the electrode surface. To verify this, the wetting behaviors of both the precursor solution and castable PCCE on the surface of the electrode material are shown in Supplementary Fig. 9 and studied. The liquid state static contact angle of the precursor solution was 82.5°, which was reduced to 67.7° after heating at 55 °C for 30 min. The reduction in the contact angle indicated that the PCCE in the castable state successfully penetrated into the electrode material. In addition, as shown in Fig. 5b, c and Supplementary Fig. 10, the electrode–electrolyte after heat treatment was more tightly bonded together by interfacial interactions. The thermal-induced phase transition of ST in the PCCE from a rigid crystalline state to a flexible viscous state led to its excellent shape adaptability and adhesion with the electrode surface. After the system returned to room temperature, the ST would crystallize again, thereby forming a mechanical interlock with the electrode surface^52^; thus, the above process strengthened the electrode–electrolyte interaction. Carbon cloth treated with polytetrafluoroethylene and a 90° peeling mode was used in viscosity tests of the PCCE and a conventional hydrogel (Fig. 5e). The toughness of the interface between the PCCE and the carbon material was 327 J m^−2^, while the interface toughness of the conventional hydrogel and the carbon material was only 24 J m^−2^. In addition, there was almost no interface toughness between the interface of the conventional inorganic solid electrolyte and the carbon material. In this work, a solid–solid interface with strong interfacial toughness was formed via a solid–liquid phase transition of phase-change materials. It had better interfacial contact between the electrode and electrolyte than the conventional solid electrolyte due to strong interfacial toughness, which also lowered the interfacial resistance. The above results were also demonstrated by an EIS analysis of the capacitor before and after heat treatment (Fig. 5e). Since the two supercapacitors differed only in the presence or absence of a heat treatment, the electrical series resistance (ESR) represented by the intersection with the real axis in the high frequency region was mainly related to electrode–electrolyte interfacial resistance. The ESR of the recrystallized electrolyte after heat treatment was much smaller than that of the electrolyte without heat treatment, and was even slightly better than that of the liquid electrolyte (Fig. 5f). This proved that the phase-transition process, which was unique to the PCCE in this work, could greatly improve the interfacial contact between the solid electrolyte and the electrode. The tough interface and the intrinsic mechanical flexibility of the electrolyte allowed the supercapacitors based on such electrolytes to have electrochemical stability under external stress. For example, a supercapacitor based on a PCCE worked normally and illuminated an LED bulb at different bending angles (Supplementary Movie 2). As shown in Fig. 5g and Supplementary Fig. 11, the CV curves and the GCD curves coincided well at different bending angles, and the capacitance remained substantially unchanged. The EIS test (Supplementary Fig. 12) proved that the internal impedance did not change significantly. In addition, we also tested the electrochemical performance of capacitors after different bending cycles. The GCD test showed that the capacitor still retained 94.1% of its initial specific capacitance after 200 bending cycles (Fig. 5h). It could also be seen from the CV (Supplementary Fig. 13) and EIS tests (Supplementary Fig. 14) that the electrochemical performance of the flexible supercapacitor remains stable after multiple bending cycles.

### Thermal-shock resistances of the PCCE-based supercapacitor

**Fig. 6:**
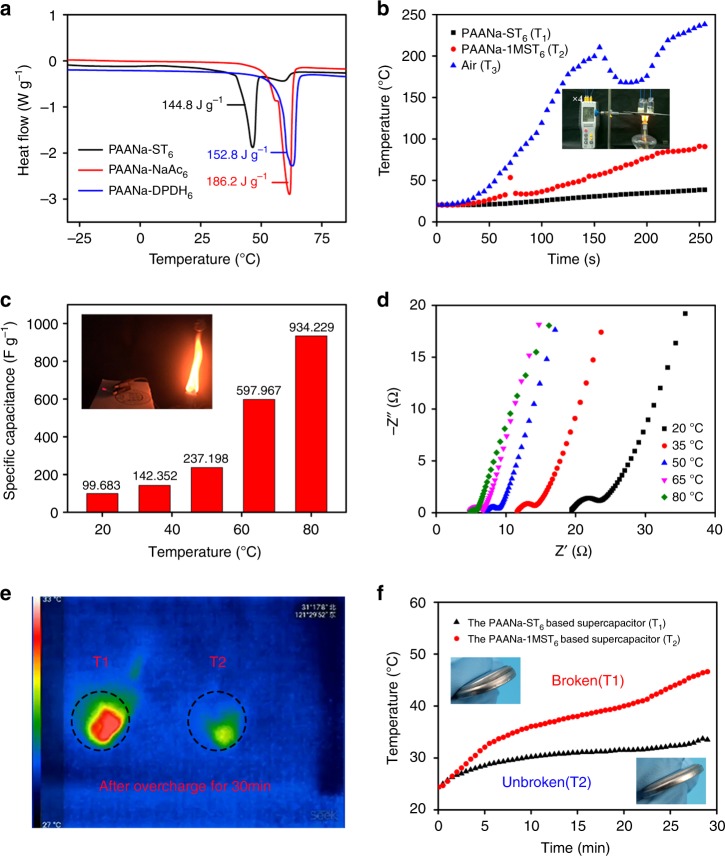
Thermal-shock resistances of the PCCE-based supercapacitor. **a** DSC curves of different PCCEs. **b** Temperature variation comparison of different PCCEs in the fire. **c** Specific capacitance of the PAANa-ST_6_-based supercapacitor in the range of 20 to 80 °C. Illustration: optical image of the PAANa-ST_6_-based supercapacitor in fire. **d** Nyquist plots of the PAANa-ST_6_-based supercapacitor in the range of 20–80 °C. **e** Thermal imaging pictures of the PAANa-ST_6_- and PAANa-1MST_6_-based supercapacitor after overcharge for 30 min at a current density of 5 A g^−1^. **f** Temperature variation comparison of PAANa-ST_6_- and PAANa-1MST_6_-based supercapacitor. Illustration: optical image of the PAANa-ST_6_- and PAANa-1MST_6_-based supercapacitor after overcharge.

More importantly, the PCCE based on the crystallohydrate–polymer composite had both high latent heat and thermal conductivity. The phase-change enthalpy was measured by a DSC test (Fig. 6a). The enthalpy values of the prepared crystallohydrate–polymer composite materials based on ST, DPDH, and NaAc were 144.8, 152.8, and 186.2 J g^−1^, respectively. The above results showed that the PCCE based on the crystallohydrate–polymer composites exhibited an excellent tolerance towards extreme temperatures. At the same time, the thermal conductivity of the ST and PAANa-ST_6_ samples was measured by a transient hot-wire method. The thermal conductivity of the sample was significantly improved by the addition of PAANa (from 0.1458 to 0.581 W m^−1^ K^−1^). When exposed to a high-temperature heat source, the hydrated salt changed from a solid state to a liquid state. As the dissolution process of the hydrated salt crystals was endothermic, the internal temperature of the crystallohydrate–polymer composites slowly increased. The presence of the polymer in the composite material enabled the liquid hydrated salt to effectively solidify, thus providing a self-supporting effect that avoided the leakage problem of conventional solid–liquid phase-change materials. To further clarify this feature, the temperature changes of a normal PAANa gel electrolyte with 1 M ST, a PCCE based on PAANa-ST_6_, and ambient air were measured in a fire using a thermocouple thermometer. After heating for 4 min, the temperature of the PAANa-ST_6_ sample was only ~40 °C, which was much lower than that of ambient air temperature (over 200 °C) and the normal PAANa gel electrolyte (~100 °C) (Fig. 6b and Supplementary Movie 3). Thus, the PAANa-ST_6_ electrolyte effectively improved the thermal resistance of the energy storage device to high temperatures. The electrochemical performance of the PAANa-ST_6_-based supercapacitor was further tested at high temperatures in consideration of the endothermic phase-transition properties of the hydrated salt component in the PCCE. As shown in Supplementary Fig. 15, the supercapacitor could work well between 20 and 80 °C, while the supercapacitor based on the normal liquid electrolyte could not work at temperatures higher than 60 °C. This was because the crystallohydrate phase-changed material could increase the boiling point of the mixture. Compared with its performance at room temperature (20 °C), the specific capacitance at 2.0 A g^−1^ increased dramatically from 99.6 to 934.2 F g^−1^ at 80 °C (Fig. 6c). The total ion concentration and ionic activity of ST were due to its solubility and the high temperature, which resulted in a high ionic conductivity and specific capacitance. In addition to the abovementioned similar factors, the better electrochemical performance was also attributed to the decrease in the strength of the PCCE at high temperatures (Supplementary Fig. 3); the reduced strength reduced the diffusional resistance during ion migration. An EIS analysis of the capacitor at different temperatures further confirmed that the higher the temperature was the lower the resistance and the higher the ionic conductivity (Fig. 6d). Moreover, a thermal cycling test was conducted in a temperature range of −20–80 °C to evaluate the thermal reliability of the PAANa-ST_6_ sample. As shown in Supplementary Fig. 16, the DSC curve showed that the phase-transition enthalpy of the composite material only changed slightly before and after the thermal cycling test (from 144.8 to 142.5 J g^−1^ after 100 thermal cycles). CV and GCD tests (Supplementary Figs. 17 and 18) before and after the thermal cycling of the capacitor based on the PCCE indicated that the electrochemical behaviors of the capacitor did not change significantly, and 94.6% of the initial specific capacitance could be retained after 100 thermal cycles. The phase-change behaviors of PAANa-ST_6_ were stable during 100 thermal cycles, and the supercapacitor based on PAANa-ST_6_ had stable electrochemical performance and good repeated resistance to extreme heat. Furthermore, we found that supercapacitors based on phase-change electrolytes had a detrimental effect on internal heat production. Through thermal imaging during the overcharge process (Fig. 6e, Supplementary Fig. 19, and Supplementary Movie 4), we could easily see that the temperature of the capacitor based on the traditional gel electrolyte had been significantly improved, while the temperature of the capacitor based on the PCCE was basically unchanged after being overcharged for 30 min at a current density of 5 A g^−1^. To further explore the ability of the PCCE to resist the internal heating of the energy storage device during overcharge, we added insulating cotton and used a thermocouple to monitor the temperature in real time during the overcharge test. As shown in Fig. 6F and Supplementary Movie 5, after overcharging for 30 min, the temperature difference of the capacitors based on different electrolytes reached ~13 °C. It was worth noting that the end of the test was due to the explosion of the capacitor based on the conventional gel electrolyte. The above result also proved that the PCCE electrolyte supercapacitor had excellent safety performance. In summary, the endothermic effect of the phase-change electrolyte could inhibit the significant increase in temperature and effectively improved the thermal-shock resistance of the energy storage device, even under fire condition (>200 °C) (Supplementary Movie 6 and illustration of Fig. 6c).

## Discussion

In summary, we prepared a series of crystallohydrate–polymer composites by a simple thermal-mixing method. Inspired by bone microstructures, the intermediate water layers between hydrated salts and polymer chains contribute to both the high mechanical strength and large ionic conductivity of the composite material. Our composite electrolytes definitely broke through the restrictive relation of strength and ionic mobility of traditional solid electrolytes. The high operating voltage and the faradic capacitance of a thiosulfate electrolyte can significantly improve the energy density and electrochemical performance of assembled supercapacitors. The reversible melting–crystallization transition of our composites can benefit the printing of solid electrolytes in various shapes, while still providing a tight assembly in flexible devices. The endothermic effect of the phase-change electrolyte can relieve the thermal-shock damage of our all-solid supercapacitors during 30 min overcharge operations. Overall, our phase-change composite, which demonstrate electrolytes high-strength, electrochemical performance and thermal resistance provide a new platform for the development of flexible and safe electric devices.

## Methods

### Electrode and electrolyte preparation

PAANa, ST pentahydrate, DPDH, NaAc, and polytetrafluoroethylene preparation (PTFE, 60 wt%) were purchased from Aladdin Industrial Co., Ltd, activated carbon powder (YP 80F, 2100 m^2^ g^−1^) was purchased from Kuraray Co., Ltd, and acetylene black was purchased from Shanghai 3F New Material Co., Ltd.

The electrodes mixture was prepared with 80 wt% active carbon, 10 wt% PTFE, and 10 wt% acetylene black. Then the mixture was spread on a titanium stainless steel collector. Last, the electrodes with mass loading of about 4 mg cm^−2^ were obtained after being dried in a vacuum at 120 °C for 24 h. Typically, the PCCE was prepared as follows: First, the ST pentahydrate was heated at 55 °C to self-dissolve. Then, the precursor was fully mixed with PAANa powder at mass ratios of 2, 4, 6, 8, and 10 for 1 min and heated at the same temperature for 1 h to obtain the PCCE samples in the castable state. The composite electrolyte with the ST self-dissolving solution began to crystallize under the perturbation of surface pressure by glass rod. Finally, the phase transition finished and a PCCE was formed with parallel stalactite-like ST crystals. The PAANa-DPDH and PAANa-NaAc samples were obtained by the same process but ST was replaced with DPDH and NaAc, respectively. 2032-type coin cells were assembled in the atmosphere.

### Materials characterization

Scanning electron microscope (Hitachi S-4800, JEOL, Tokyo, Japan) was used to observe the surface structure of the composite freeze-dried samples at a voltage of 3 kV. The phase-change composites were prepared as a film shape, and were measured by an X-ray powder diffractometer (D8 advance, Bruker). The FT-IR spectra were recorded on a Thermo Scientific Nicolet IS10 with wavenumbers ranging from 4000 to 400 cm ^−1^ with KBr powder at a resolution of 0.4 cm^−1^.

### Electrochemical measurements

The ionic conductivity of the composite materials was measured according to a previously described method^46^. The energy density, power density, and specific capacitance of supercapacitors are calculated as described in the supporting information^53^. The ionic conductivity of pure ST pentahydrate crystals was monitored using an HQ30d portable meter, Hach, USA.

### Other characterizations

DSC scans were conducted on an Instruments RCS90 scanning calorimeter at a heating rate of 5 °C min^−1^. The temperature of the different systems was measured using a thermocouple thermometer (HT-9815, Dongguan Xintai Instrument Co., Ltd). The compressive stress–strain properties of the composite materials were measured using an electronic universal testing machine (UTM2502, Shenzhen Suns Technology Co., Ltd). Compressive tests of the composite samples, were carried out at a speed of 2 mm min^−1^ with 95% strain. The combined energy of the water was compared by nuclear magnetic resonance analyzer (PQ001-20-025, Suzhou Niumag Analytical Instrument Co., China) at room temperature. 3D printing was done with an extrusion 3D printer (Bioscaffolder 3.2, GeSiM, Germany) at 80 °C.

### Reporting summary

A reporting summary for this article is available as a Supplementary Information file.

## Supplementary information

Supplementary Information

Description of Additional Supplementary Files

Supplementary Movie 1

Supplementary Movie 2

Supplementary Movie 3

Supplementary Movie 4

Supplementary Movie 5

Supplementary Movie 6

## Data Availability

Additional data related to this paper may be requested from the corresponding authors (W.Q. (wangqg66@tongji.edu.cn) or W.B. (wangbaofeng@shiep.edu.cn)).
