# Supplementary material for: Water-mediated crystallohydrate–polymer composite as a phase-change electrolyte
Source: Nat Commun. 2020 Apr 15;11:1843. doi: 10.1038/s41467-020-15415-5 (PMC7160156; doi:10.1038/s41467-020-15415-5)
Supplement: Supplementary file 1 — Supplementary Information [file 41467_2020_15415_MOESM1_ESM.pdf]

Supporting Information for

Water-Mediated Crystallohydrate-Polymer Composite as a Phase-Change Electrolyte

Tai. et. al.

Supplementary Figures

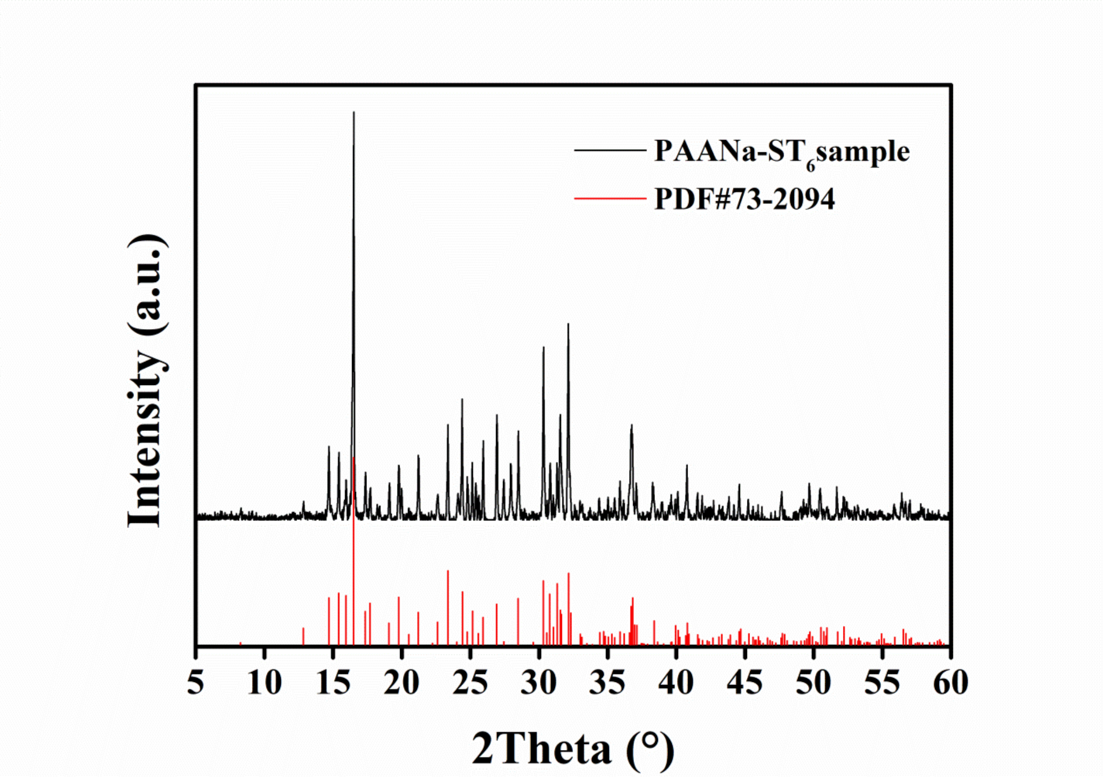

Supplementary Figure 1. XRD patterns of PAANa-ST_6 sample.

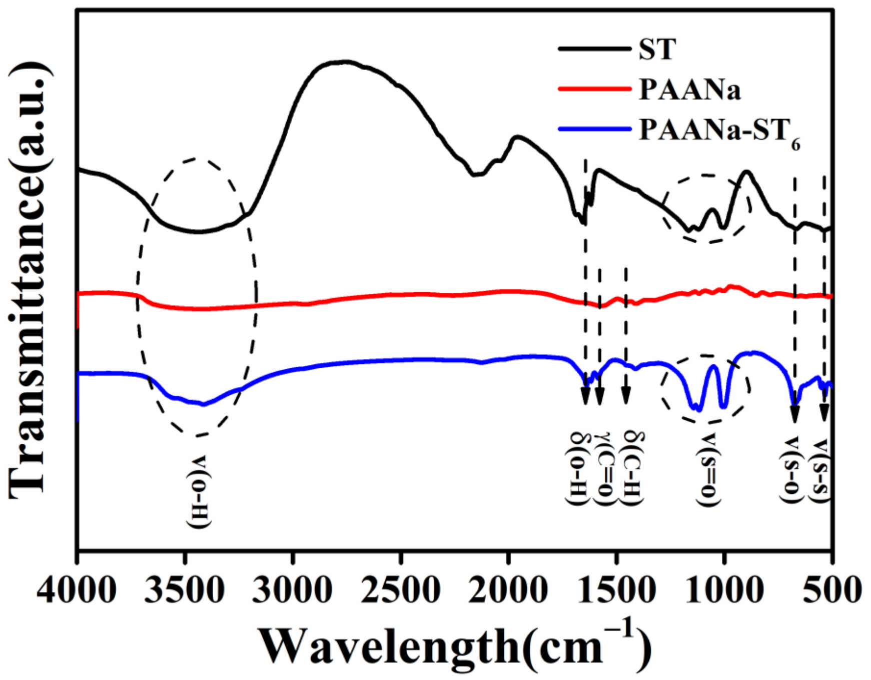

Supplementary Figure 2. FT-IR patterns of ST, PAANa, and PAANa-ST_6 samples.

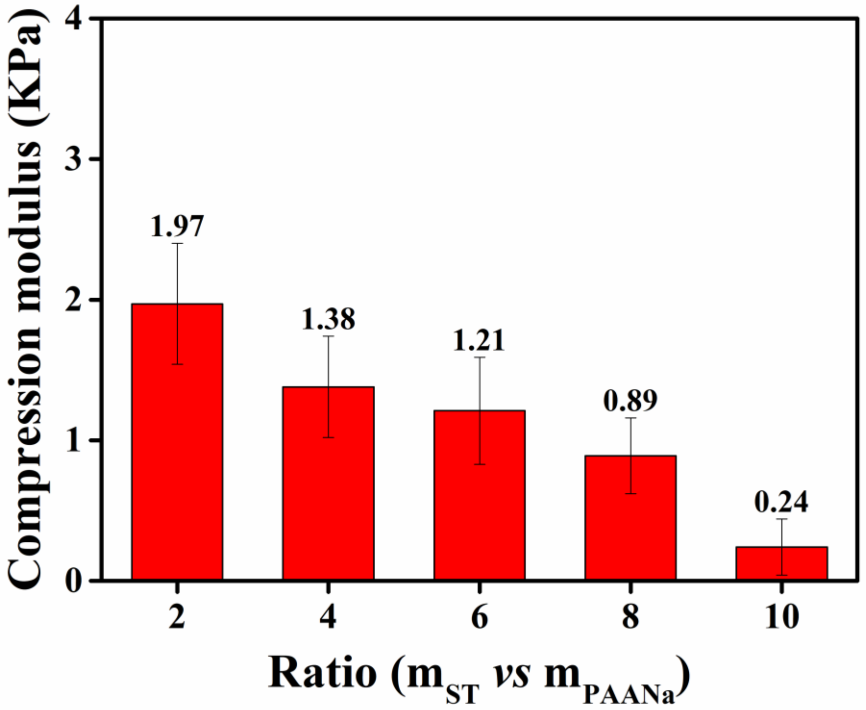

Supplementary Figure 3. Compressive curves of the composite electrolytes with different mass ratios of ST in castable state.

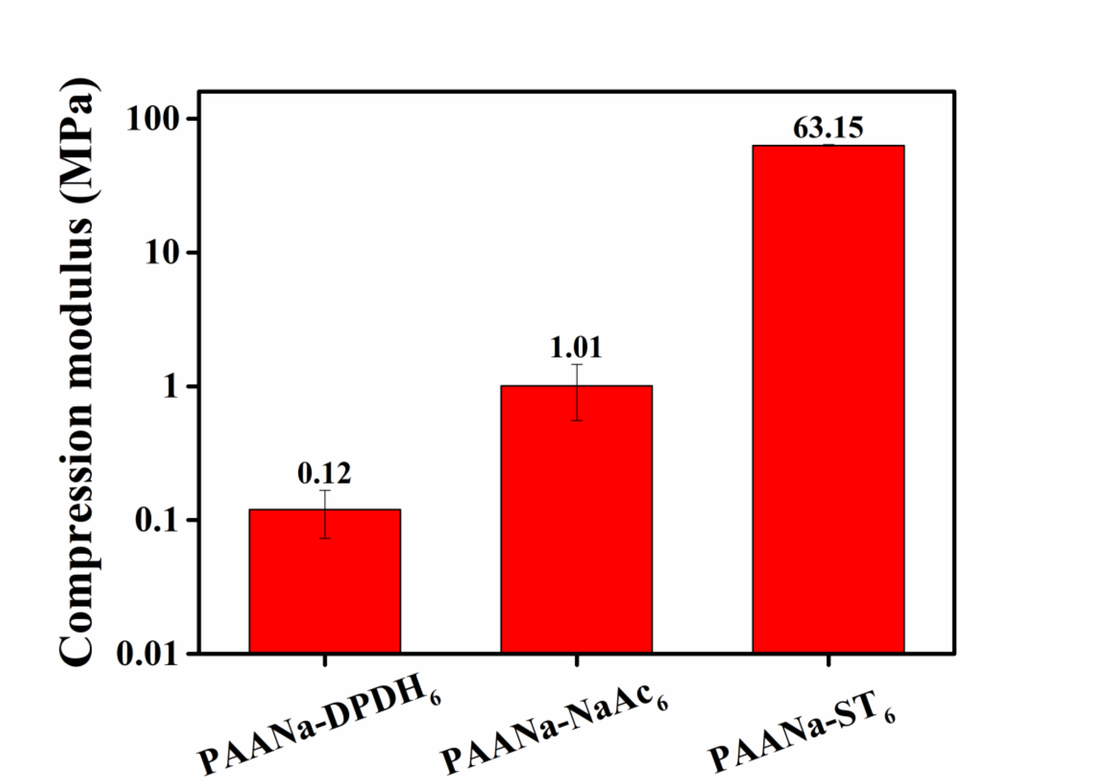

Supplementary Figure 4. Compression modulus of PAANa-DPDH$_6$, PAANa-NaAc$_6$ and PAANa-ST$_6$ samples.

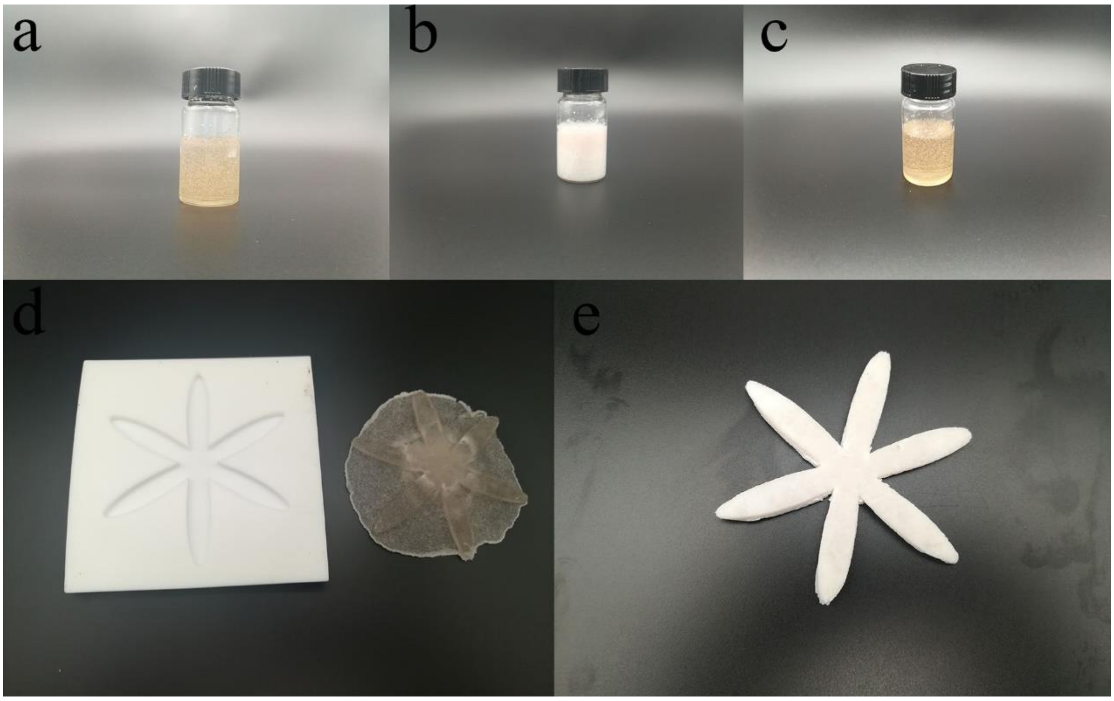

Supplementary Figure 5. Demonstration of plasticity properties. **a**–**c** The optical image of the composite electrolyte freely converted between rigid (crystalline state) and flexible (castable state) in high temperatures and room temperature. **d**–**e** The optical image of reprocessing of the composite electrolyte.

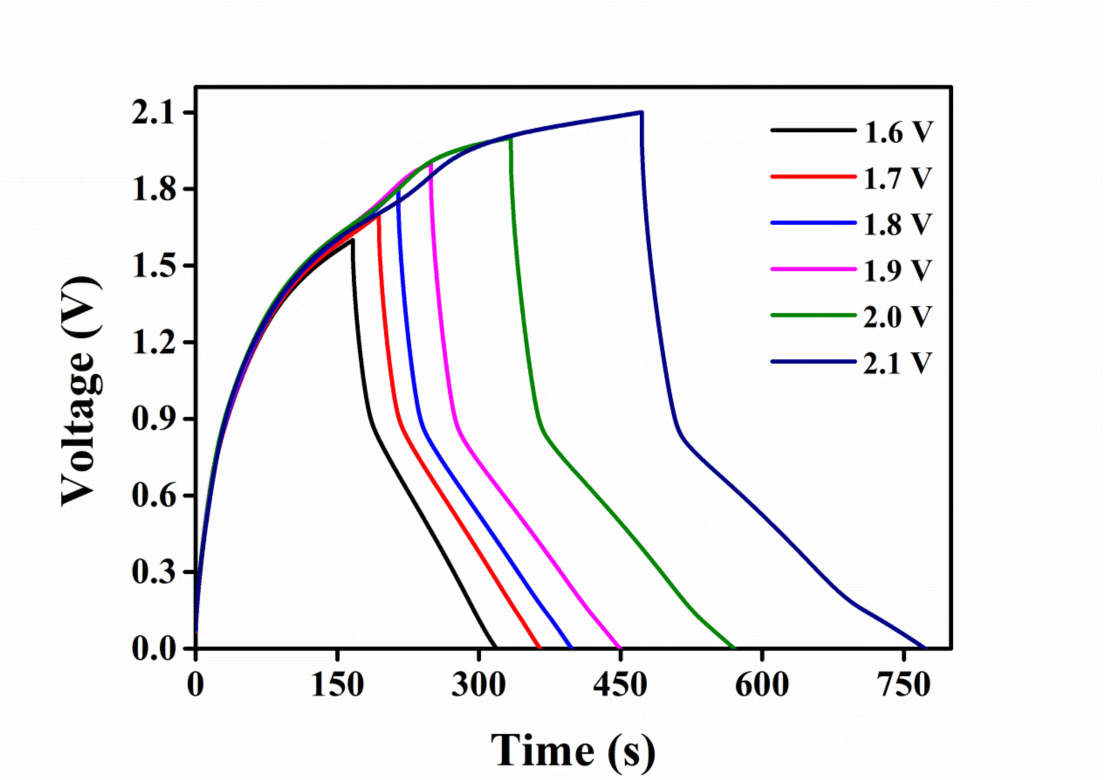

Supplementary Figure 6. Galvanostatic charge-discharge curves of the PAANa-ST_6-based supercapacitor with different cut-off voltages from 1.6 V to 2.1 V.

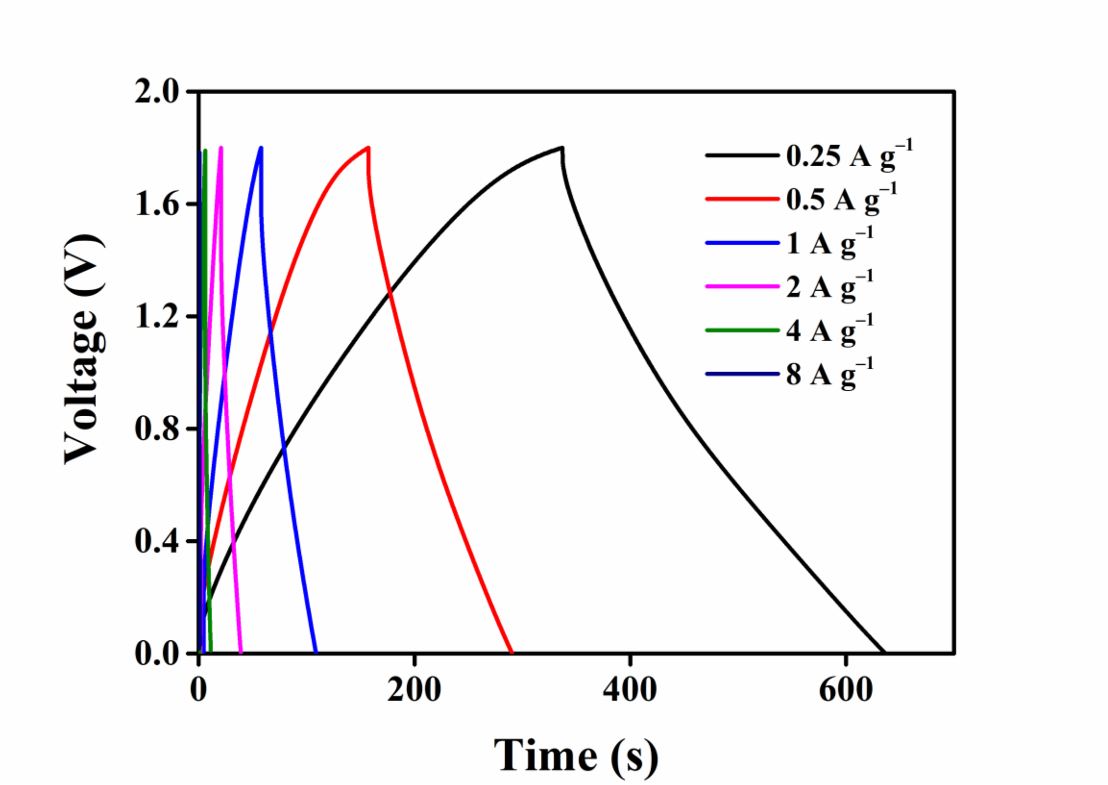

Supplementary Figure 7. Galvanostatic charge-discharge curves of the PAANa-DPDH$_6$-based supercapacitor.

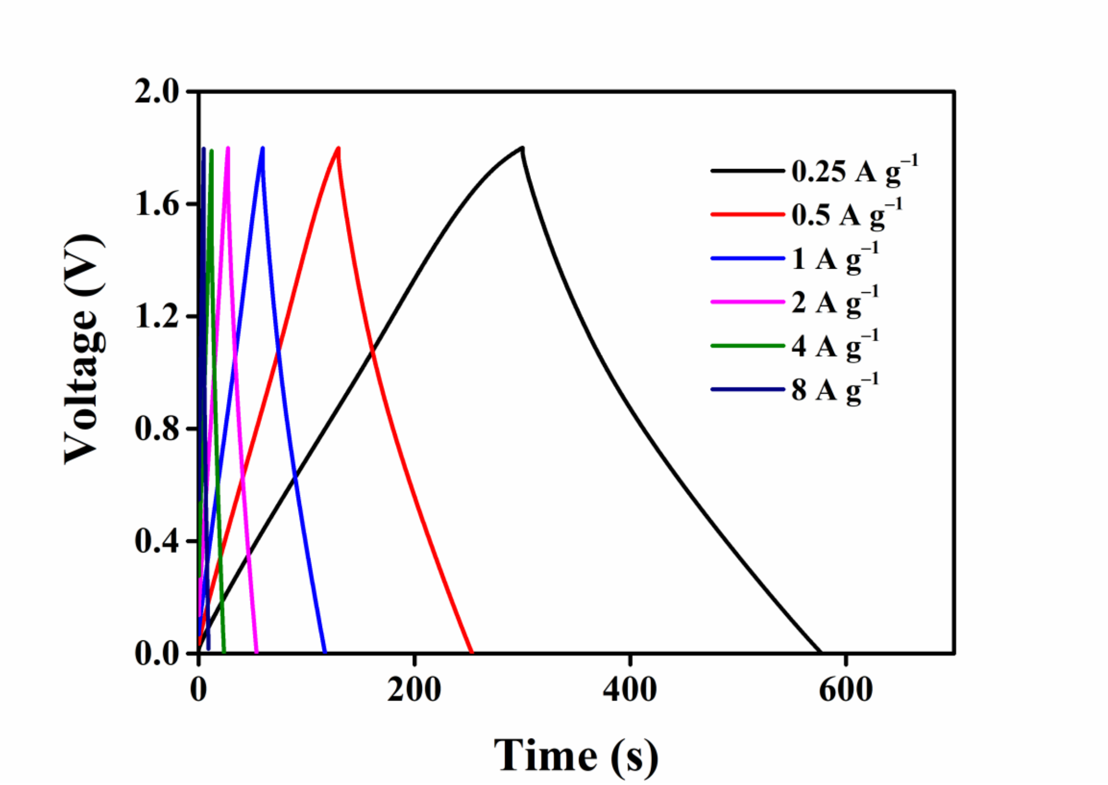

Supplementary Figure 8. Galvanostatic charge-discharge curves of the PAANa-$NaAc_6$-based supercapacitor.

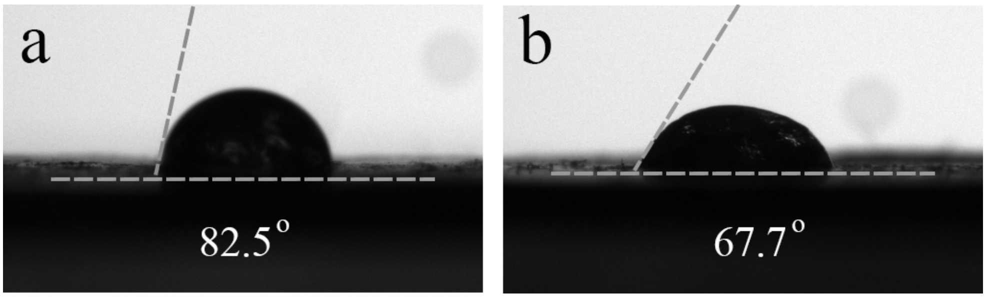

Supplementary Figure 9. Contact angles analysis. a Static contact angles of precursor solution and **b** castable PAANa-ST6 sample on the surface of electrode.

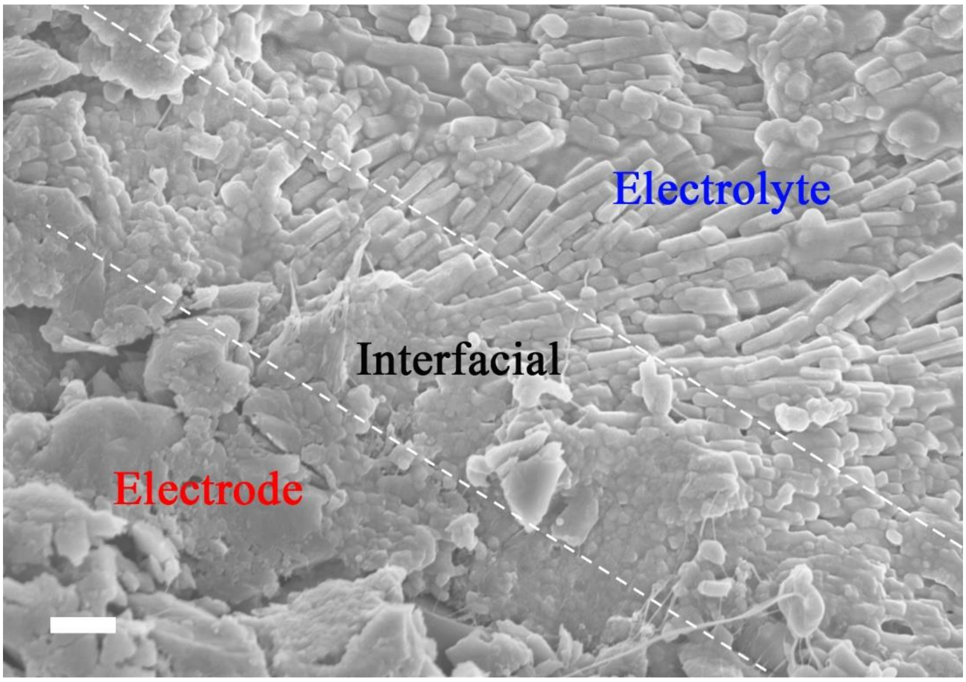

Supplementary Figure 10. Scanning electron microscope of the interface between electrode and electrolyte in the capacitor after heat treatment. Scale bar, 1 μm.

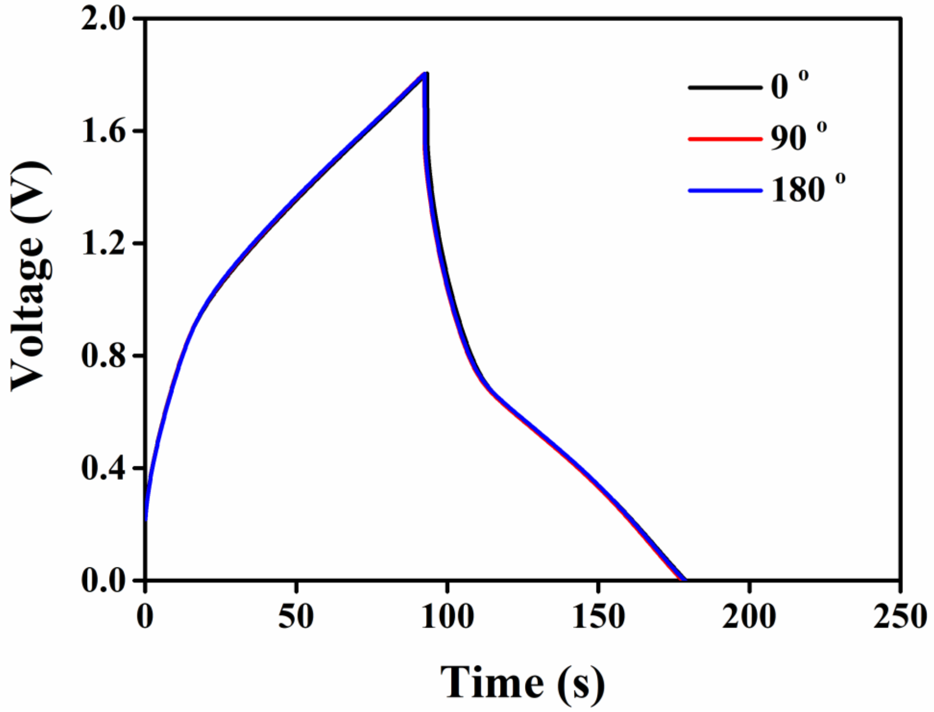

Supplementary Figure 11. Galvanostatic charge-discharge curves of the PAANa-ST_6-based supercapacitor at different bending angles.

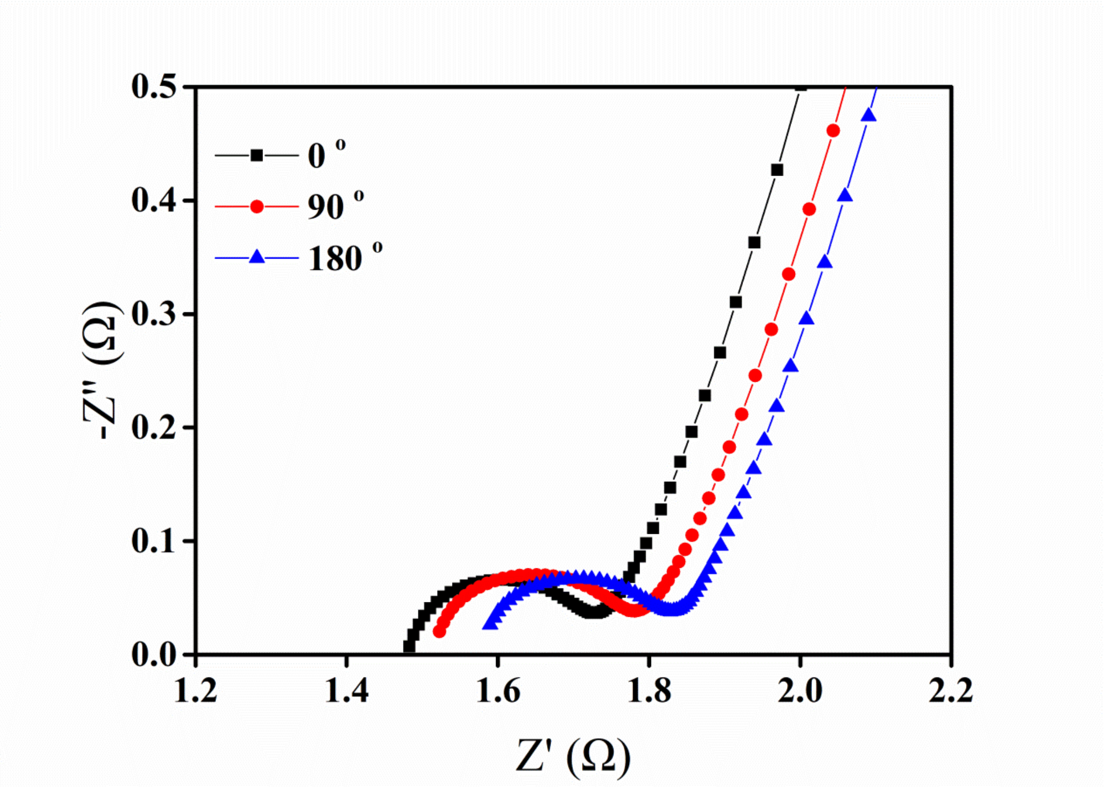

Supplementary Figure 12. Electrochemical impedance spectroscopy of the PAANa-ST_6-based supercapacitor at different bending angles.

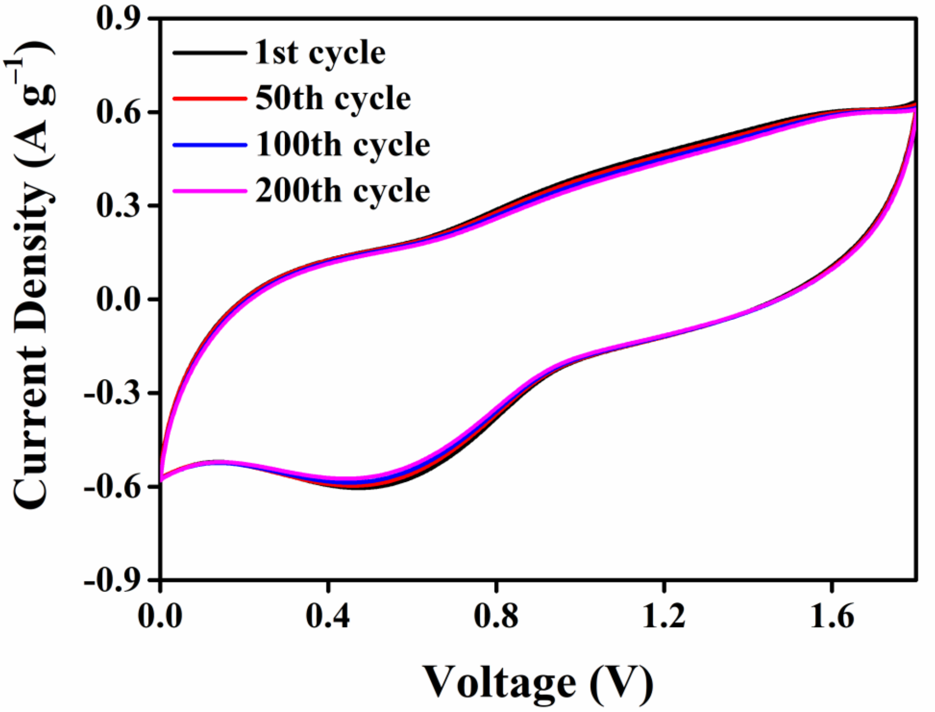

Supplementary Figure 13. Cyclic voltammetry of the PAANa-ST_6-based supercapacitor after different bending cycles.

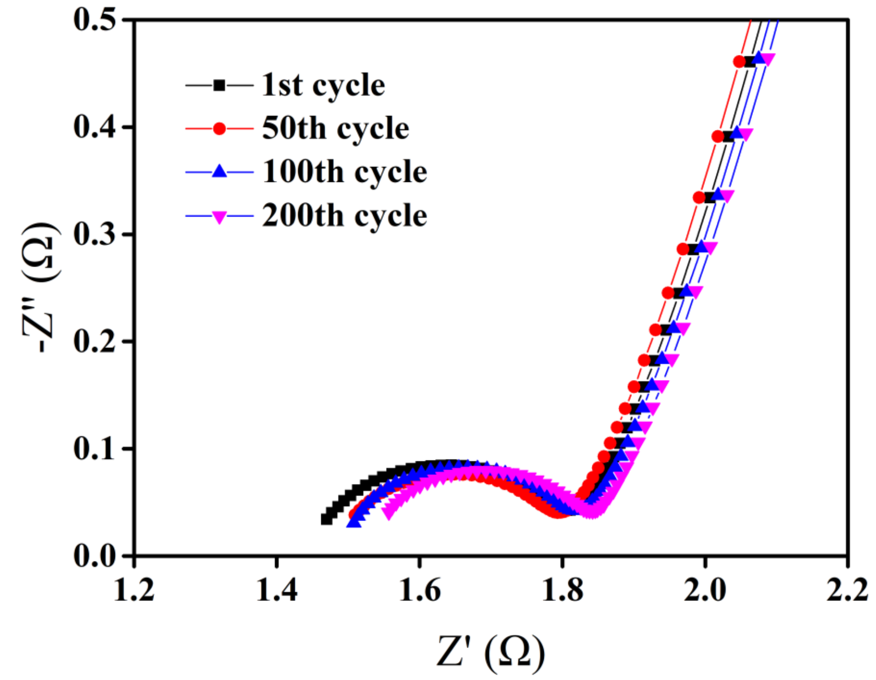

Supplementary Figure 14. Electrochemical impedance spectroscopy of the PAANa-$NaAc_6$-based supercapacitor at different bending angles.

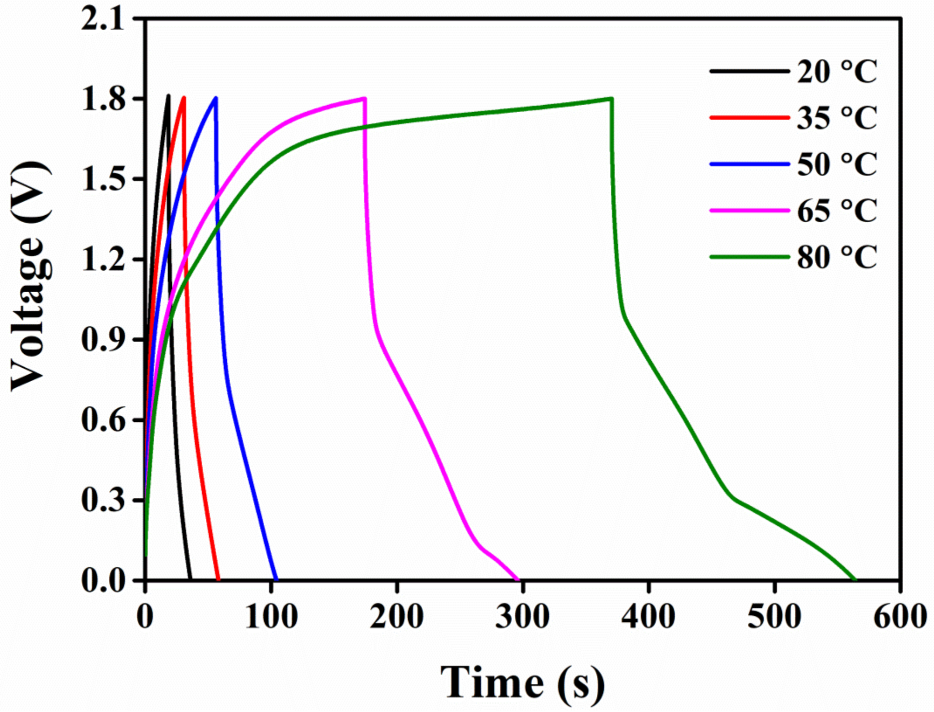

Supplementary Figure 15. Galvanostatic charge-discharge curves of the PAANa-ST$_6$-based supercapacitor at a current density of 2 A g^{-1} in the range of 20 °C to 80 °C.

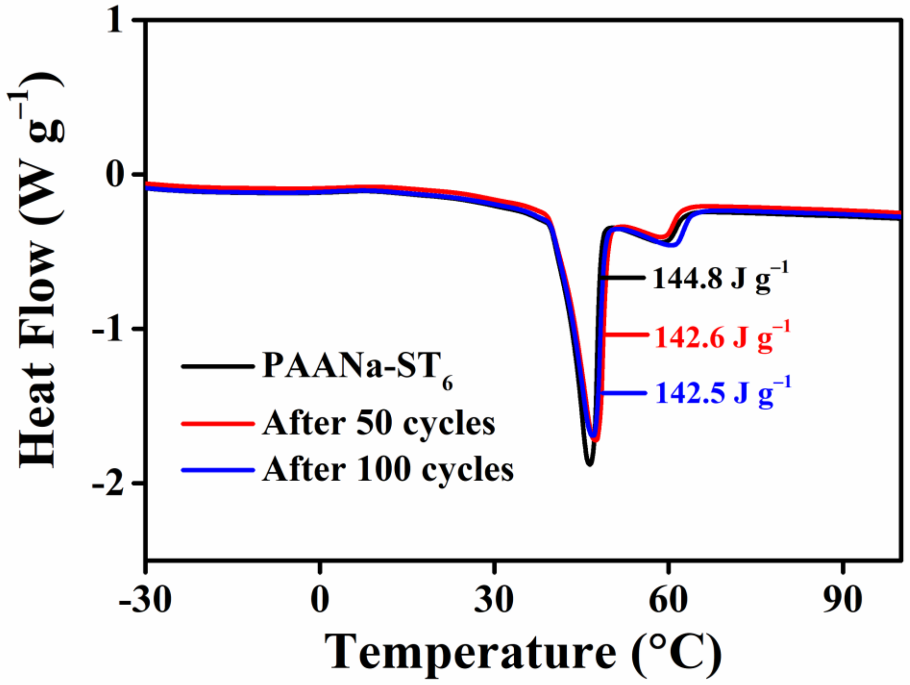

Supplementary Figure 16. DSC curves of PAANa-ST$_6$ composite before and after thermal cycling test.

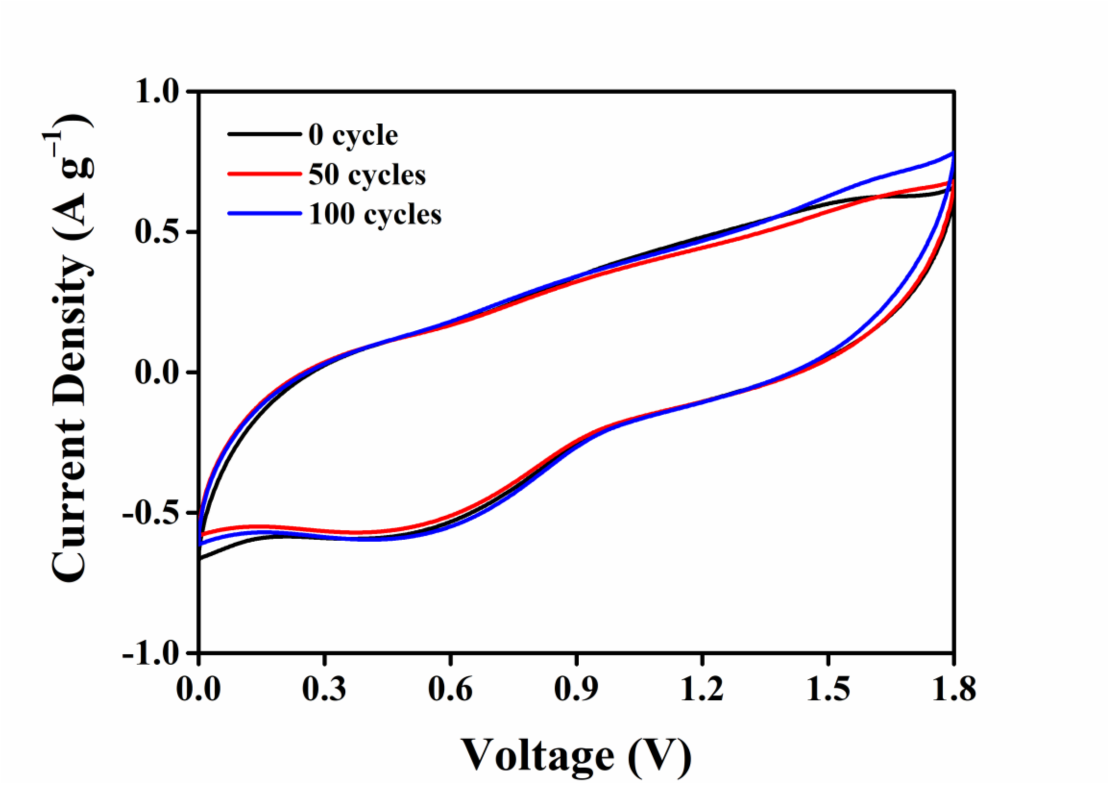

Supplementary Figure 17. Cyclic voltammetry curves of the PAANa-ST_6-based supercapacitor before and after thermal cycling test.

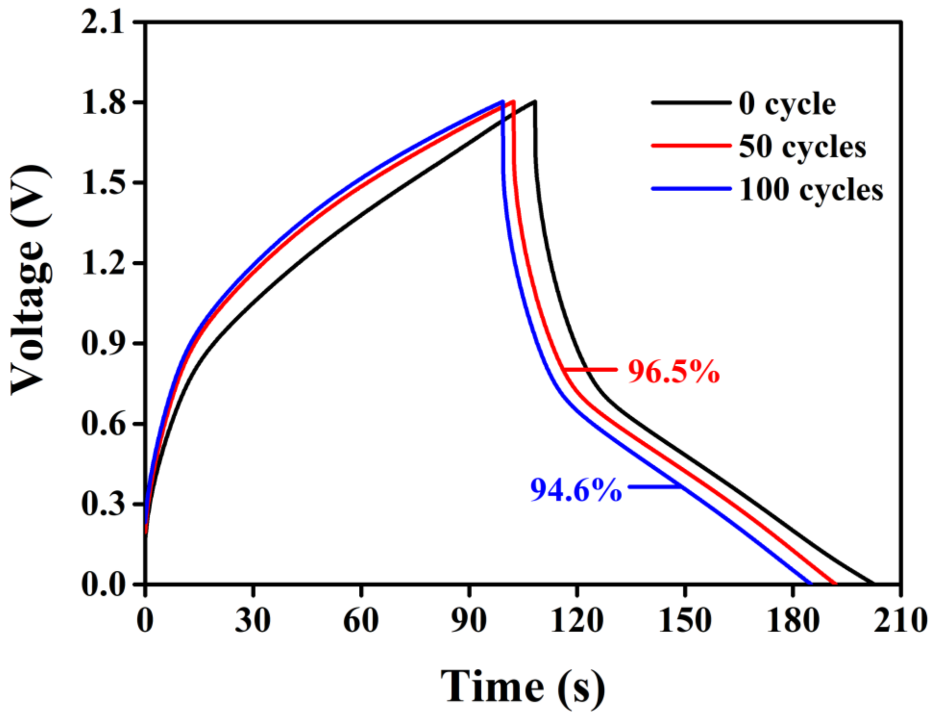

Supplementary Figure 18. Galvanostatic charge-discharge curves of the PAANa-ST_6-based supercapacitor before and after thermal cycling test.

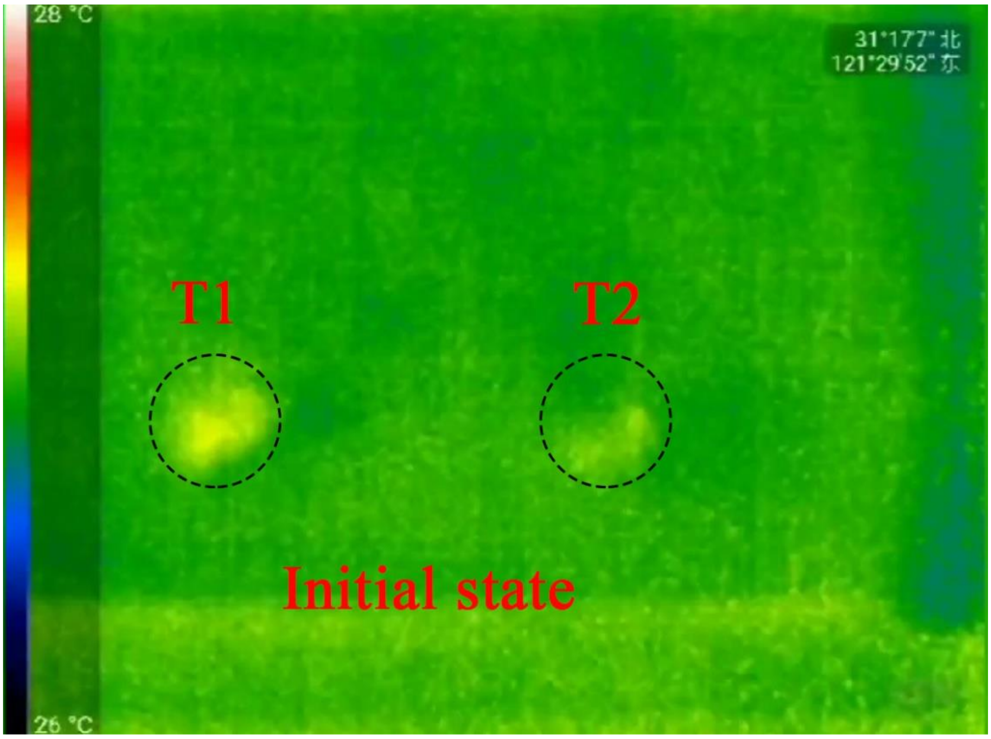

Supplementary Figure 19. Thermal imaging pictures of the PAANa-ST_6 and PAANa-1MST_6 based supercapacitor in initial state.

Supplementary Tables

Supplementary Table 1. The water-lose energy of sodium thiosulfate and the water-obtained energy by sodium acrylate at 55 °C.

**n**	**ΔG(55 °C) / eV**		
	The energy required for sodium thiosulfate to lose the nth water		The energy released for sodium polyacrylate to obtains the nth water.
1st	0.38	<	0.64
2nd	0.41	>	0.39
3rd	0.43	>	0.37
4th	0.59	>	0.36
5th	0.70	>	0.02

Supplementary Table 2. The interaction of water or sodium with sodium thiosulfate or sodium acrylate at 25 °C.

	**G_{int}(25 °C) / eV**	
	Interaction of water with sodium thiosulfate or sodium acrylate	Interaction between Na^+ and sodium thiosulfate system or sodium acrylate system
$Na_2S_2O_3 \cdot 5H_2O$	0.48	6.56
$Na_2S_2O_3 \cdot 4H_2O$	0.51	6.34
$(C_3H_3O_2Na)_2 \cdot H_2O$	0.45	6.30
$(C_3H_3O_2Na)_2$	/	6.34

Supplementary Methods

Electrochemical measurements

The energy density, power density and specific capacitance of the supercapacitor were calculated as follows:

The specific capacitance C_S can be calculated from the galvanostatic charge-discharge curves as follow equation:

$$C_S = \frac{4It}{mV} \tag{1}$$

Where I is the discharge current, t is the discharge time, m is the active material mass of the electrode, V is the voltage after IR drop.

The energy density and power density are calculated using the following equations:

$$E = \frac{C_S V^2}{8} \tag{2}$$

$$P = \frac{E}{t} \tag{3}$$

Where E is the energy density, C_S is the specific capacitance calculated from equation 1, V is the voltage after IR drop, P is power density and t is the discharge time.

Theoretical Calculations

All calculations were performed with the Gaussian 09 program1, and the B3LYP/6-311+g(d, p) method was used to optimize geometric structures. The model of PAANa was composed of two monomers to matching the same positive and negative groups with $Na_2S_2O_3{\cdot}5H_2O$. The free energy difference in exchange of water, ΔG is calculated following Supplementary Eq. 4:

$$\Delta G = G_0(H_2O) + G_0(M{\cdot}n\text{-}1H_2O) - G_0(M{\cdot}nH_2O) \quad (4)$$

M is $Na_2S_2O_3$ or PAANa. The interaction free energy, G_{int}, of the interaction intensity between the water or Na^+ and $Na_2S_2O_3$ or PAANa in the system is calculated following Supplementary Eqs. 5 and 6:

$$G_{int}(H_2O) = [G_0(M\ nH_2O) + n\times G_0(H_2O) - G_0(M)]/n \quad (5)$$

$$G_{int}(Na) = G_0([M{-}Na]\ nH_2O) + G_0(Na^+) - G_0(M{\cdot}nH_2O) \quad (6)$$

Based on the above definition, a negative G_{int} corresponds to stable interaction between the components, and the more postive G_{int} indicates a stronger interaction in the system.
