# Supplementary material for: Water-mediated crystallohydrate–polymer composite as a phase-change electrolyte
Source: Nat Commun. 2020 Apr 15;11:1843. doi: 10.1038/s41467-020-15415-5 (PMC7160156; doi:10.1038/s41467-020-15415-5)
Supplement: Supplementary file 2 — Description of Additional Supplementary Files [file 41467_2020_15415_MOESM2_ESM.pdf]

Description of Additional Supplementary Files

File Name: Supplementary Movie 1

Description: The crystallization process of the self-dissolving ST solution in the composite.

File Name: Supplementary Movie 2

Description: Display of flexible supercapacitors lighting LED bulb at different bending angles.

File Name: Supplementary Movie 3

Description: Demonstration of temperature rise of different types of electrolytes under flame burning conditions.

File Name: Supplementary Movie 4

Description: Thermal imaging movie showing the heating process of different types of supercapacitors under overcharge conditions.

File Name: Supplementary Movie 5

Description: Demonstration of the actual heating speed of different types of supercapacitors under overcharge conditions.

File Name: Supplementary Movie 6

Description: Display of PCCE-based supercapacitors lighting LED bulb under flame burning conditions.
